# Burden, Management, and Outcome of Needle-Stick Injuries Among Healthcare Workers at Muhimbili National Hospital in Dar es Salaam, Tanzania

**DOI:** 10.7759/cureus.111678

**Published:** 2026-06-28

**Authors:** Layla M Babiker, Essam Arabi, Omer I Idriss, Ahmed I Idriss, Mohamed Elobaid, Mohamed M Mabuie, Elmustafa I Idriss, Osama Yasin, Ahmed Suliman, Ruba I Ahmed, Marwa M Mohammed

**Affiliations:** 1 Internal Medicine, International University of Africa, Khartoum, SDN; 2 Internal Medicine, Sudan International University, Khartoum, SDN; 3 General Internal Medicine, University Hospital Kerry, Tralee, IRL; 4 Internal Medicine, University of Medical Sciences and Technology, Khartoum, SDN; 5 Internal Medicine, University of Khartoum, Khartoum, SDN; 6 Surgery, University of Khartoum, Khartoum, SDN; 7 General Surgery, Cavan Monaghan General Hospital, Cavan, IRL; 8 Medicine, King Salman Hospital, Riyadh, SAU; 9 Medical Education, University of Medical Sciences and Technology, Khartoum, SDN; 10 Pediatrics, University of Khartoum, Khartoum, SDN

**Keywords:** healthcare workers, hepatitis b, hepatitis c, human immunodeficiency virus, needle-stick injuries, occupational safety and health administration

## Abstract

Introduction

Needle-stick injuries (NSIs) are a major occupational hazard for healthcare workers (HCWs), exposing them to blood-borne pathogens such as human immunodeficiency virus (HIV), hepatitis B virus (HBV), and hepatitis C virus (HCV). Despite existing prevention protocols, NSIs remain common in resource-limited settings. This study assessed the burden, management, and outcomes of NSIs among HCWs at Muhimbili National Hospital, Dar es Salaam, Tanzania.

Methods

A descriptive cross-sectional study was conducted among 150 healthcare workers from various professional categories. Data were collected from January to December 2025 using a structured self-administered questionnaire assessing demographics, NSI exposure, reporting behavior, post-exposure management, knowledge of NSI protocols, preventive practices, psychological impact, and HBV vaccination status. Descriptive statistics were used to summarize the findings.

Results

Eighty participants (53.3%) reported experiencing at least one NSI during their career. Among exposed participants, 33 (41.3%) reported experiencing two to three NSIs within the previous 12 months. The most common circumstances of injury were patient procedures (n=34, 43.0%), disposal of sharps (n=16, 20.3%), and needle recapping (n=13, 16.5%). Most participants were aware of hospital NSI protocols (n=134, 89.3%) and had received formal training (n=107, 71.3%); however, 68 participants (45.4%) reported always or sometimes recapping needles. Reporting rates were high, with 69 participants (87.3%) reporting their most recent NSI. Post-exposure prophylaxis (PEP) was received or offered to 61 participants (77.2%), and 38 participants (51.4%) initiated PEP within two hours of exposure. Psychological distress following NSI was reported by 67 participants (87.0%). Hepatitis B vaccination coverage was 68.7% (n=103), while 24 participants (16.0%) remained unvaccinated.

Conclusion

NSIs remain a significant occupational challenge at Muhimbili National Hospital, with substantial prevalence, recurrent injuries, and persistent unsafe practices. Although reporting systems and post-exposure response mechanisms appear robust, gaps remain in needle-handling practices and HBV vaccination coverage. Strengthening preventive training, enforcing non-recapping policies, expanding access to safety-engineered devices, and improving vaccination coverage may help reduce occupational exposure among HCWs.

## Introduction

Needle-stick injuries (NSIs) are among the most important occupational hazards encountered by healthcare workers (HCWs). These injuries occur when needles, scalpels, blades, scissors, or other sharp medical devices accidentally penetrate or cut the skin. Although the resulting wounds are often minor, exposure to contaminated instruments can place HCWs at risk of contact with blood and other potentially infectious body fluids. Consequently, NSIs represent a significant route of occupational exposure to blood-borne pathogens in healthcare settings [1].

The occurrence of NSIs remains a global concern. Data from 52 Ministry of Health hospitals in the Kingdom of Saudi Arabia demonstrated an annual sharp injury rate of 3.2 injuries per 100 occupied hospital beds. In that study, nurses were the most frequently affected group, hospital wards were the most common sites of injury, disposable syringes were the devices most often involved, and many injuries occurred during routine clinical use [1]. Comparable reports from the United States have shown considerably higher rates, reaching 20.7 injuries per 100 occupied beds in teaching hospitals and 16.5 injuries per 100 occupied beds in non-teaching hospitals [2,3].

NSIs pose a serious threat because they facilitate the transmission of numerous blood-borne infections. More than 20 infectious pathogens have been associated with occupational exposure through NSIs, with human immunodeficiency virus (HIV), hepatitis B virus (HBV), and hepatitis C virus (HCV) being the most clinically significant [3]. Beyond the risk of infection, these incidents may result in substantial psychological distress among affected HCWs, including anxiety, depression, fear of disease acquisition, and reduced quality of life. The estimated risk of transmission following percutaneous exposure is approximately 0.2% for HIV, 1.8% for HCV, and as high as 30% for HBV. Of these major blood-borne infections, HBV remains the only one that can be effectively prevented through vaccination [4].

Despite advances in infection prevention strategies, including staff education, safer device design, and improved disposal systems, NSIs continue to occur throughout the handling, use, disassembly, and disposal of sharp instruments. The Occupational Safety and Health Administration (OSHA) estimates that approximately 5.6 million HCWs in the United States are at risk of occupational exposure to blood-borne pathogens through such injuries [4]. NSIs have been recognized as a significant occupational hazard across a wide range of healthcare professions, including dental and allied health personnel. Globally, an estimated three million of the approximately 35 million HCWs experience occupational exposure to blood-borne pathogens through NSIs each year [5]. In Africa, factors such as work experience, long working hours, job dissatisfaction, inadequate use of personal protective equipment, poor adherence to infection prevention guidelines, and lack of infection prevention training have been identified as important contributors to NSI occurrence [5].

## Materials and methods

Study design

A cross-sectional facility-based study was conducted at Muhimbili National Hospital (MNH) in Dar es Salaam, Tanzania, using quantitative data collection approaches.

Study area

The study was conducted at MNH, the largest national referral and teaching hospital in Dar es Salaam. The hospital employs approximately 2,700 HCWs, including about 300-340 doctors and specialists, 900-970 nurses, and other supporting staff. MNH has a bed capacity of approximately 1,500 and provides care to an estimated 1,000-1,200 outpatients and 1,000-1,200 inpatients per week.

Study population

The study population consisted of HCWs at MNH, including doctors, nurses, and laboratory technicians. All HCWs at MNH, including medical staff, nurses, and laboratory technicians, were eligible for inclusion in the study. There were no exclusion criteria applied in this study.

Sampling technique and sample size

A simple random sampling technique was used between January and December 2025 to enrol participants from the eligible HCW population at MNH. The sample size was determined using Cochran’s formula, where n₀ = Z²pq/d². In this formula, Z was set at 1.96, p at 0.5, q at 0.5, and d at 0.05. Based on this calculation, the estimated sample size was approximately 150 healthcare workers.

Data collection, management, and statistical analysis

Data were collected using a structured questionnaire administered to HCWs at MNH. Each participant was assigned a unique identification code to ensure confidentiality and secure handling of data throughout the study. The collected data were manually entered into the IBM SPSS Statistics for Windows, Version 25 (Released 2017; IBM Corp., Armonk, New York, United States) and subjected to double-entry verification to minimize transcription errors. Responses with missing or unclear information, such as “Prefer not to say,” were coded and treated as missing values during analysis. Since all study variables were categorical in nature, tests of normality were not required. Descriptive statistical analysis was performed using frequencies and percentages for all variables. The results were then summarized and presented using tables and charts to describe demographic characteristics, the burden of NSIs, management practices, and outcome-related variables.

## Results

Sociodemographic characteristics

Doctors made up the largest group of participants (n=47, 31.3%), followed by nurses (n=40, 26.7%), medical students (n=26, 17.3%), laboratory technicians (n=23, 15.3%), and others (n=14, 9.3%). Most respondents had one to five years of healthcare experience (n=85, 56.7%), while 20.7% (n=31) had less than one year, 12.7% (n=19) had six to 10 years, and 10% (n=15) had more than 10 years of experience. Participants were distributed across several departments, with Surgery (n=26, 17.3%) and Obstetrics & Gynecology (n=26, 17.3%) being the most represented, followed by Internal Medicine (n=25, 16.7%), Pediatrics (n=23, 15.3%), Laboratory (n=19, 12.7%), Emergency (n=19, 12.7%), and others (n=12, 8.0%) (Table 1).

**Table 1:** Sociodemographic characteristics of healthcare workers at the Muhimbili National Hospital

Parameter		Frequency (n)	%
Current professional role	Doctor	47	31.3%
	Nurse	40	26.7%
	Laboratory technician	23	15.3%
	Medical student	26	17.3%
	Others	14	9.3%
Years of experience in the healthcare field	Less than 1 year	31	20.7%
	1-5 years	85	56.7%
	6-10 years	19	12.7%
	More than 10 years	15	10.0%
Department or unit you currently work in	Surgery	26	17.3%
	Internal medicine	25	16.7%
	Laboratory	19	12.7%
	Obstetrics & Gynecology	26	17.3%
	Pediatrics	23	15.3%
	Emergency	19	12.7%
	Others	12	8.0%

Exposure and burden

More than half of participants (n=80, 53.3%) reported experiencing an NSI at work, while 70 participants (46.7%) had not. In the past 12 months, 21.3% (n=17) had no NSI, 33.8% (n=27) experienced one NSI, 41.3% (n=33) experienced two to three NSIs, and three participants (3.8%) had more than three NSIs. The most common cause of NSI was during patient procedures (n=34, 43.0%), followed by disposal of sharps (n=16, 20.3%), recapping used needles (n=13, 16.5%), and instrument cleaning (n=12, 15.2%). A small proportion reported other causes (n=4, 5.1%), and one respondent preferred not to mention the cause (Table 2).

**Table 2:** Prevalence, frequency, and circumstances of needle-stick injuries among healthcare workers

		Frequency (n)	%
History of needle-stick injury (NSI) while at work	Yes	80	53.3%
	No	70	46.7%
How many times have you experienced a needle-stick injury in the past 12 months?	0	17	21.3%
	1	27	33.8%
	2-3	33	41.3%
	More than 3	3	3.8%
What was the most common cause of your needle-stick injury?	Recapping used needles	13	16.5%
	During disposal of sharps	16	20.3%
	During patient procedure	34	43.0%
	During instrument cleaning	12	15.2%
	Others	4	5.1%
	Prefer not to say	1	1.3%

Management & reporting

Among participants who had experienced an NSI and responded to this question (n=79), 69 (87.3%) reported their most recent NSI, while 12.7% (n=10) did not, and one person preferred not to answer. Among those who did not report the injury, half (n=5, 50%) believed it was a minor injury, and two (20%) were too busy or lacked time, 30% (n=3) selected other reasons, and one preferred not to say. No participant reported fear of blame or lack of knowledge of procedures. Among respondents who answered this question (n=79), 61 (77.2%) received or were offered post-exposure prophylaxis (PEP), while 14 respondents (17.7%) did not, and four respondents (5.1%) were unsure. Among those who started PEP, 51.4% (n=38) began within two hours, 31.1% (n=23) within two to 24 hours, 2.7% (n=2) after 24 hours, 14.9% (n=11) were not sure, and six preferred not to say (Table 3).

**Table 3:** Reporting practices and post-exposure management following needle-stick injuries among healthcare workers

Parameter		Frequency (n)	%
Did you report your most recent needle-stick injury to your supervisor or relevant authority?	Yes	69	87.3%
	No	10	12.7%
	Prefer not to say	1	1.3%
If you did not report, what was the main reason?	Fear of blame or punishment	0	0.0%
	Believed it was a minor injury	5	50.0%
	Didn’t know reporting procedures	0	0.0%
	Too busy / lack of time	2	20.0%
	Others	3	30.0%
	Prefer not to say	1	9.1%
Were you offered or did you receive post-exposure prophylaxis (PEP) following the injury?	Yes	61	77.2%
	No	14	17.7%
	Not sure	4	5.1%
	Prefer not to say	1	
How soon after the injury did you start PEP (if applicable)?	Within 2 hours	38	51.4%
	2-24 hours	23	31.1%
	After 24 hours	2	2.7%
	Not Sure	11	14.9%
	Prefer not to say	6	

Knowledge & prevention

Most participants (n=134, 89.3%) were aware of the hospital’s protocol for managing NSIs, while 16 participants (10.7%) were unaware. About 71.3% (n=107) had received formal training on NSI prevention and management, whereas 28.7% (n=43) had not. Personal protective equipment (PPE) use was high, with 72.7% (n=109) always using PPE when handling sharps, 18.7% (n=28) sometimes, 7.3% (n=11) rarely, and two participants (1.3%) never using PPE. Regarding recapping practices, 38.0% (n=57) never recapped needles, while 26.7% (n=40) always recapped, 18.7% (n=28) sometimes recapped, and 16.7% (n=25) rarely recapped them (Table 4).

**Table 4:** Knowledge of needle-stick injury protocols and preventive practices among healthcare workers

Parameter		Frequency (n)	%
Are you aware of the hospital’s protocol or guidelines for managing needle-stick injuries?	Yes	134	89.3%
	No	16	10.7%
Have you received formal training on needle-stick injury prevention and management?	Yes	107	71.3%
	No	43	28.7%
How often do you use personal protective equipment (PPE) when handling sharps or needles?	Always	109	72.7%
	Sometimes	28	18.7%
	Rarely	11	7.3%
	Never	2	1.3%
How often do you recap used needles after use?	Always	40	26.7%
	Sometimes	28	18.7%
	Rarely	25	16.7%
	Never	57	38.0%

Outcome & psychological impact

Among respondents who answered this question (n=77), 67 (87.0%) experienced anxiety, stress, or fear following their most recent NSI, while 13.0% (n=10) did not; three participants preferred not to answer. Among respondents who answered this question (n=78), 65 (83.3%) were tested for HIV and/or HBV following the injury, eight respondents (10.3%) were not tested, five respondents (6.4%) were unsure, and two preferred not to say. Among those tested, 60.0% (n=39) were tested for both HIV and HBV, 36.9% (n=24) for HIV only, and 3.1% (n=2) for HBV only. Regarding HBV vaccination status, 68.7% (n=103) were fully vaccinated, 10.0% (n=15) were partially vaccinated, 16.0% (n=24) were not vaccinated, and 5.3% (n=8) were unsure (Table 5).

**Table 5:** Psychological impact of needle-stick injuries and Hepatitis B vaccination status among healthcare workers

Parameter		Frequency (n)	%
Did you experience anxiety, stress, or fear after your most recent needle-stick injury?	Yes	67	87.0%
	No	10	13.0%
	Prefer not to say	3	
Were you tested for HIV and/or Hepatitis B following the injury?	Yes	65	83.3%
	No	8	10.3%
	Not Sure	5	6.4%
	Prefer not to say	2	
If you were tested, specify which one	HIV only	24	36.9%
	HBV only	2	3.1%
	Both HIV and HBV	39	60.0%
Hepatitis B vaccination status	Fully vaccinated	103	68.7%
	Partially vaccinated	15	10.0%
	Not vaccinated	24	16.0%
	Not sure	8	5.3%

## Discussion

This study assessed the burden, management, and outcomes of NSIs among healthcare workers at the MNH. Overall, NSIs remain an important occupational hazard, with 53.3% (n=80) of respondents reporting at least one NSI during their career. This finding confirms that tertiary hospitals with high patient throughput continue to face substantial occupational exposure risk [1,3].

A notable proportion of exposed respondents (41.3%, n=33) experienced two to three NSIs within the past 12 months, indicating recurrent exposures rather than isolated events. Recurrent injuries have been reported in several settings and are often associated with high workload, inadequate staffing, and repeated performance of invasive procedures [1,4]. Recurrent NSIs increase cumulative risk of pathogen exposure and highlight the need for interventions that address systemic and workflow factors, including duty hours, staffing levels, and task distribution.

In our sample, the most frequent circumstances of injury were during patient procedures (43.0%, n=34), followed by disposal of sharps (20.3%, n=16) and needle recapping (16.5%, n=13). This distribution is consistent with multiple studies showing that device use and procedure-related handling account for the majority of NSIs [1,3]. Importantly, although 89.3% (n=134) of participants reported awareness of hospital protocols and 71.3% (n=107) had received formal training, unsafe practices persisted, with 45.4% reporting “always” or “sometimes” recapping needles (26.7%, n=40 always; 18.7%, n=28 sometimes). This disconnect between knowledge and practice echoes findings from other investigations and systematic reviews, which show that knowledge alone does not reliably translate into safer behavior without continuous supervision, reinforcement, and availability of safety-engineered devices [3,6].

The psychological impact of NSIs was substantial, with 87.0% (n=67) of exposed participants reporting anxiety, stress, or fear following the injury. This emotional burden is well described in the literature and can have downstream effects on staff wellbeing and performance [4,6]. The high rate of testing after exposure (83.3%, n=65 tested for HIV and/or HBV) and the relatively prompt initiation of PEP are encouraging from a clinical-management perspective, but mental health support and clear counseling pathways should be incorporated into institutional post-exposure protocols [5].

Reporting behavior in this study was relatively high, with 87.3% (n=69) of those injured reporting the most recent event. This contrasts with many reports of pervasive underreporting in similar settings, where barriers such as fear of blame, uncertainty about procedures, and perceived triviality of the injury reduce reporting rates [4,7]. In our data, the main reason for not reporting was the belief that the injury was minor (50.0%, n=5) rather than fear of blame, suggesting that reporting systems may be accessible and non-punitive but that educational messaging should emphasize that all exposures merit reporting and follow-up.

High reporting rates likely contributed to the comparatively good post-exposure management noted here, as 77.2% (n=61) received or were offered PEP and over half initiated PEP within two hours (51.4%, n=38), aligning with recommended practice and comparing favorably with several regional reports [5,8]. A further 31.1% (n=23) initiated PEP within two to 24 hours, while 2.7% (n=2) started after 24 hours.

HBV vaccination coverage in this cohort was moderate, with 68.7% (n=103) fully vaccinated, 10.0% (n=15) partially vaccinated, and 16.0% (n=24) unvaccinated. Although this compares favorably with some settings, it leaves a significant minority unprotected against a preventable occupational risk [1,9]. Ensuring complete HBV vaccination for all clinical staff remains a priority and can substantially reduce long-term morbidity associated with occupational exposures [10].

Taken together, these findings suggest a mixed but actionable picture: strong institutional practices in reporting and PEP availability coexist with persistent unsafe handling behaviors, particularly needle recapping, and incomplete immunization coverage. Practical implications include prioritizing targeted behavior-change interventions that go beyond didactic training, such as simulation, on-the-job coaching, and audit-feedback systems; removing systemic workflows that encourage recapping or unsafe disposal; improving access to safety-engineered devices and adequate sharps containers at points of care; and strengthening universal HBV vaccination programs for all at-risk staff [11,12].

The psychological and emotional consequences of NSIs have also been emphasized in occupational health literature, reinforcing the importance of institutional psychosocial support systems and structured counseling pathways following exposure [13,14].

Comparable prevalence estimates from pooled and regional analyses further support the persistence of occupational exposure in healthcare settings, particularly in low- and middle-income countries [15]. Likewise, favorable post-exposure management outcomes observed in this study are consistent with regional findings reporting timely PEP uptake and appropriate follow-up among exposed healthcare workers [16].

Strengths of this study include the inclusion of multiple healthcare professional categories (doctors, nurses, laboratory staff, and students), use of a structured instrument with double data-entry verification, and reporting of both behavioral and outcome variables, including reporting rates, PEP timing, psychological impact, and vaccination status. Limitations should also be acknowledged. The cross-sectional design precludes causal inference, and the data were self-reported and therefore subject to recall and social desirability bias, potentially leading to overreporting of protocol awareness or PEP uptake. Furthermore, the sample was drawn from a single tertiary hospital, which may limit generalizability to other settings. Finally, although the study captured frequency and proximate causes of NSIs, it was not designed to measure device-level factors, such as the availability of safety-engineered needles, or to perform multivariable risk-factor analysis that are important areas for future research.

## Conclusions

NSIs remain a common occupational hazard among HCWs at the MNH, with more than half of participants reporting at least one lifetime exposure and many experiencing recurrent injuries. Although reporting practices and post-exposure management systems were generally satisfactory, persistent needle recapping, incomplete HBV vaccination coverage, and the psychological impact of exposure continue to present important occupational health concerns.

Strengthening infection prevention strategies through regular practical training, strict adherence to non-recapping policies, wider availability of safety-engineered devices, comprehensive post-exposure support, and universal HBV vaccination programs may substantially reduce occupational exposure risks and improve HCW safety.
